# Using Advanced Diffusion-Weighted Imaging to Predict Cell Counts in Gray Matter: Potential and Pitfalls

**DOI:** 10.3389/fnins.2022.881713

**Published:** 2022-06-03

**Authors:** Hamsanandini Radhakrishnan, Sepideh Kiani Shabestari, Mathew Blurton-Jones, Andre Obenaus, Craig E. L. Stark

**Affiliations:** ^1^Mathematical, Computational and Systems Biology, University of California, Irvine, Irvine, CA, United States; ^2^Department of Neurobiology and Behavior, School of Biological Sciences, University of California, Irvine, Irvine, CA, United States; ^3^Department of Pediatrics, School of Medicine, University of California, Irvine, Irvine, CA, United States

**Keywords:** MRI, diffusion weighted imaging (DWI), cell count, non-invasive biomarkers, prediction model, High Angular Resolution Diffusion Imaging (HARDI), NODDI

## Abstract

Recent advances in diffusion imaging have given it the potential to non-invasively detect explicit neurobiological properties, beyond what was previously possible with conventional structural imaging. However, there is very little known about what cytoarchitectural properties these metrics, especially those derived from newer multi-shell models like Neurite Orientation Dispersion and Density Imaging (NODDI) correspond to. While these diffusion metrics do not promise any inherent cell type specificity, different brain cells have varying morphologies, which could influence the diffusion signal in distinct ways. This relationship is currently not well-characterized. Understanding the possible cytoarchitectural signatures of diffusion measures could allow them to estimate important neurobiological properties like cell counts, potentially resulting in a powerful clinical diagnostic tool. Here, using advanced diffusion imaging (NODDI) in the mouse brain, we demonstrate that different regions have unique relationships between cell counts and diffusion metrics. We take advantage of this exclusivity to introduce a framework to predict cell counts of different types of cells from the diffusion metrics alone, in a region-specific manner. We also outline the challenges of reliably developing such a model and discuss the precautions the field must take when trying to tie together medical imaging modalities and histology.

## Introduction

While advances in immunohistochemistry and microscopy have been extremely valuable in capturing microstructural properties of the brain at cellular resolution, these techniques are not very feasible in human studies, and cannot be used *in vivo* and non-invasively. Modern diffusion analysis techniques are well-equipped to non-invasively detect various aging and cognition-related microstructural properties in gray matter (Budde and Annese, 2013; Aggarwal et al., 2015; Colgan et al., 2016; Assaf, 2019). While there has been work to validate the relationship between diffusion metrics and various white matter properties (Jelescu and Budde, 2017), our understanding of what neurobiological properties they reflect in gray matter is far less developed. Moreover, the sensitivity of these diffusion metrics is yet to be taken advantage of in a predictive capacity. Examining specific structural properties across scales of measurement would not only assist in delineating changes particular to certain disease and senescence states but could also enable the identification of valid non-invasive biomarkers specific to these states.

As diffusion imaging has evolved (Wu and Miller, 2017), the need for validating and re-examining these diffusion models has only become more pressing. By taking advantage of data that includes estimates of diffusion along many directions, newer models, like those derived from High Angular Resolution Diffusion Imaging (HARDI) techniques, hold the potential for higher accuracy and specificity. However, just as older diffusion tensor techniques could largely only reliably predict white matter microstructure, most attempts to find histological correlations with diffusion metrics have primarily focused on white matter. Moreover, despite a growing number of potentially more advanced and powerful models, the diffusion tensor has remained the focus for most of these correlation studies (Table 1).

**TABLE 1:** Analysis of studies correlating tensor metrics with cellular properties.

Species	Region of interest	Observations
*Ex vivo* wild type mice (Chang et al., 2016)	Corpus callosum, fimbria, fornix	FA: Positively correlated with myelin density
*Ex vivo* rats with retinal ischemia (Rojas-Vite et al., 2019)	Optic nerve and chiasm	FA: positively correlated with axon density, volume fraction, and myelin volume fraction. Negatively correlated with axon diameter and myelin thickness.
*Ex vivo* Human with multiple sclerosis (Mottershead et al., 2003; Schmierer et al., 2007)	Whole-brain white matter, spinal cord white matter	FA: positively correlated with myelin density and axon count. MD: negatively correlated with myelin density and axon count.
*Ex vivo* human with Alzheimer’s disease (Gouw et al., 2008)	Whole-brain white matter	FA: Positive correlated with axonal density
*Ex vivo* elderly human (Back et al., 2011)	Prefrontal cortex white matter	FA: Negatively correlated with free radical injury and oligodendrocyte lineage marker. MD: Positively correlated with free radical injury, oligodendrocyte lineage marker, and myelin damage
*In vivo* human with temporal lobe epilepsy (Concha et al., 2010)	Fornix	FA: Positively correlated with total axon membrane circumference

While studies in Table 1 have been very useful in understanding standard tensor metrics like fractional anisotropy and mean diffusivity, few such studies have been conducted to correlate axonal structure measures obtained from histology with more advanced diffusion metrics, like those derived from HARDI acquisition schemes. Even fewer have attempted to study this in gray matter, even though these more recent diffusion metrics may be more effective at examining gray matter microstructure (Radhakrishnan et al., 2020, 2021; Venkatesh et al., 2020).

Moreover, the nomenclature of these diffusion metrics can be misleading or vague. For example, NDI stands for “neurite density index,” yet is designed simply to measure intracellular volume fraction, while the brain has more cell types than just neurons. Glial cells contribute significantly to diffusion metrics, but this is often overlooked to simplify the model. Correspondingly, another NODDI metric, the ODI, is positively correlated with microglial density (Yi et al., 2019), demonstrating the potential of these modern diffusion models to probabilistically estimate cell type-specific counts. Moreover, the different inflammatory states of astrocytes and microglia have been found to be reflected in certain biophysical model-based diffusion metrics (Garcia-Hernandez et al., 2020). Models like these can be particularly valuable when trying to track disease progression, success of interventions or the extent of injuries.

3D-BOND (3D Bridging of Optically clear histology with Neuroimaging Data) is one of the only pipelines developed for registering medical images with 3D histology, with a focus on bridging the gap between meso-resolution MRI and cellular-resolution microscopy (Stolp et al., 2018). This study not only showed that, within the mouse hippocampus, axonal content was correlated with apparent fiber density (AFD), mean diffusivity (MD), and radial diffusivity (RD) in a 3D space, but it also demonstrated that metrics like MD and RD were associated with cell density. Even more specifically, FA was observed to be positively correlated with astrocyte density, suggesting that diffusion metrics had the potential to garner information beyond just white matter integrity. In fact, diffusion metrics have successfully been shown to be sensitive to a variety of neurobiological changes outside of white matter, like effects of immunosuppression on aging (Radhakrishnan et al., 2021), developmental consequences of neuronal apoptosis in cortical regions (Petrenko et al., 2018) and the maturation pattern of deep gray matter regions like the caudate nucleus during healthy development (Pietsch et al., 2019). Studies like these are extremely valuable as they help translate between microstructural diffusion tensor metrics and distinct cellular properties. However, such studies often depend on simple linear regression models when comparing diffusion metrics with cellular properties, while it is likely that more mathematically complex models may better represent these relationships. Furthermore, as we will demonstrate, different regions may present very different relationships between these metrics and cellular properties, but the brain’s complex spatial dynamics are often understandably overlooked to generate potentially less accurate but simpler whole-brain models.

In this paper, we outline a framework to non-invasively predict cell counts using diffusion metrics, after considering the challenges discussed above. We first show that a single model is not capable of capturing the morphological complexity of the whole brain, and that different regions have different diffusion metric/cell count relationships. We then successfully develop an algorithm that can separately predict the counts of different cell types in an individual region. We picked the CA1 of the hippocampus as the test region to demonstrate this, given its appropriate level of cytoarchitectural complexity and size.

## Materials and Methods

### Animals

All animal procedures were conducted in accordance with the guidelines set forth by the National Institutes of Health (NIH) and the University of California, Irvine Institutional Animal Care and Use Committee. All mice were age and sex-matched and group-housed on a 12 h/12 h light/dark cycle with food and water *ad libitum*. Six B6CBAF1/J mice (Jackson Laboratory, stock number 100011) were perfused at P120 with ice-cold 1× PBS. Brains were in 4% PFA for 48 h and then stored in 1× PBS until MRI scanning.

### MR Image Acquisition

**FIGURE 1:**
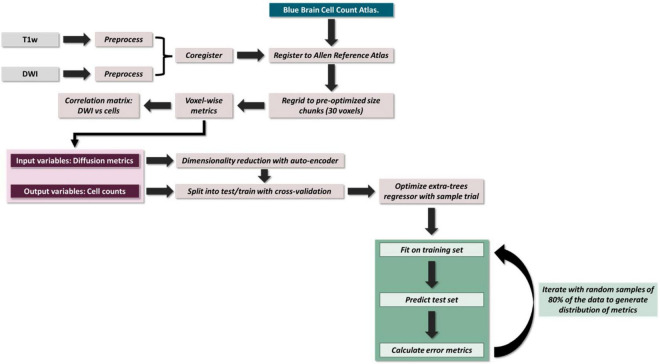
Overview of pipeline.

The brains were scanned in skull *ex vivo* using an Avance III HD spectrometer manufactured by Bruker Bio-Spin operating at a field strength of 17.6 T (750 MHz) with an 89 mm bore (Figure 1). The temperature in the scanner was between 21 and 22°C.

**T1w:** A Fast Low Angle Shot (FLASH) scan was acquired with the following parameters: echo time (TE)/repetition time (TR) = 20/160 ms, flip angle = 30°, and in 0.07 mm isotropic resolution.

**DWI:** Coronal diffusion-weighted echo-planar images were acquired with b = 1,000 s/mm^2^ (20 directions) and b = 3,000 s/mm^2^ (52 directions) with the following parameters: TE/TR = 5/28 s, FOV = 212 × 182 mm, pulse duration –4 ms, pulse spacing = 12 ms and in 0.125 mm isotropic resolution. Two images with no diffusion weighting (b = 0) were also collected.

### Diffusion Preprocessing

All preprocessing steps employed MRtrix3 (Tournier et al., 2012)^1^ commands or used MRtrix3 scripts that linked external software packages. Image denoising was first performed by using a random matrix theory-derived threshold for PCA denoising (Veraart et al., 2016), followed by removal of Gibbs ringing artifacts (Kellner et al., 2016), eddy current correction (Andersson and Sotiropoulos, 2016), and bias field correction (Tustison et al., 2014). The image intensity was then normalized across subjects in the log-domain (Raffelt et al., 2012). Images with no diffusion weighting (*b* = 0) were extracted and averaged to aid with structural registration.

### Structural Preprocessing

Each subject’s structural image was non-linearly co-registered to the average of their respective preprocessed b0 images (ANTS v2.3.4) (Tustison et al., 2010), so that the structural and diffusion images were in the same space for the rest of the analyses. Registration was manually checked to ensure accuracy. These images were then non-linearly co-registered to the Allen 3D Reference Atlas, which had been constructed from averaging high-resolution two-photon tomography images from 1,675 young adult C57BL/6J mice (Lein et al., 2007; Wang et al., 2020).

### Deriving Diffusion Metrics

We calculated traditional tensor metrics using MRtrix3. A weighted least squares (WLS) approach was first used to fit the diffusion tensor to the log signal, using weights based on empirical signal intensities (Basser et al., 1994). We repeated the weighted least squares with weights determined by the signal predictions from the previous step (Veraart et al., 2013). We then generated maps of the following tensor-derived parameters: the mean apparent diffusion coefficient (ADC, sometimes also referred to as Mean Diffusivity or MD), fractional anisotropy (FA), axial diffusivity (AD, same as principal eigen value) and radial diffusivity (RD, equal to mean of the two non-principal eigen values) (Westin, 1997).

Higher-order multi-compartment metrics were derived using the *ex vivo* Neurite Orientation Dispersion and Density Imaging (NODDI) (Zhang et al., 2012) model in the Microstructure Diffusion Toolbox (Harms et al., 2017), and the intrinsic diffusivity was set to the default of 0.6 μm^2^ ms^–1^. Note that even though the NODDI model typically generates three primary metrics: NDI, ODI, and FISO, our analysis for this paper is limited to the NDI and the ODI. The FISO is a free water measure, typically proportional to the amount of CSF in a voxel, and such a measure is meaningless in perfused tissue.

### Deriving Cell Counts

Typical cell counts from each voxel were obtained from the Markram atlas (Erö et al., 2018). The atlas uses a variety of whole-brain image datasets, including Nissl-staining for cells and genetic marker stains to distinguish glia from neurons, as well as subtype staining for both glia (astrocytes, microglia, and oligodendrocytes) and neurons (excitatory and inhibitory). A unique property of this atlas is that it is not limited to generating a single expected value at each location. Rather, it integrates data from the literature to be able to reflect local variation based on individual variability. Using this atlas to generate cell counts was preferred over empirically determining them not only because it eliminated most of the experimental noise and error, but also because this atlas promised more robust estimates as they were combined from multiple sources. A limitation of the atlas is that it does not take into account individual differences in cell counts of the mice imaged in this study.

### Voxel-Wise Correlations

**FIGURE 2:**
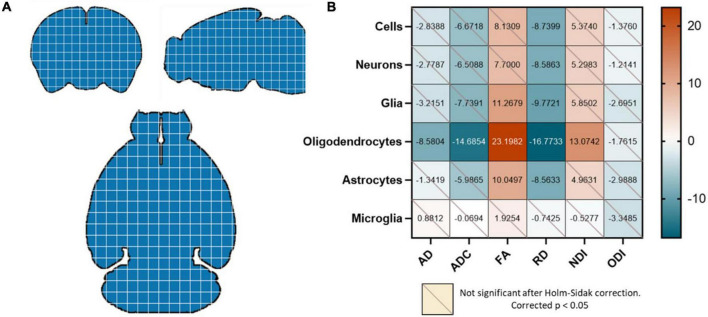
**(A)** The whole brain was re-gridded into 30 × 30 × 30 voxels, and data points were generated by averaging the diffusion metrics and cell counts in each of these voxels for each mouse. Each blue square here represents a 30 × 30 × 30 voxel that was designated a unique value. **(B)** Oligodendrocyte counts were highly correlated with all diffusion metrics except the ODI. FA was positively correlated with glia and astrocytes as well; and RD was negatively correlated with all cell types except microglia. Values in the correlation matrix represent the *t*-value from a one-sample *t*-test of the Z-score of the Pearson correlation coefficient of each subject’s pair. “Cells” represent counts of all cell types studied, and “Glia” is the sum of oligodendrocyte, astrocyte and microglia counts.

To perform voxel-wise correlations, region-specific masks were generated using the Allen 3D Reference Atlas, and the masks were eroded by a factor of 3 to account for any partial volume effects. To deal with any registration artifacts and individual differences in cellularity, these masks were then re-gridded from 0.125 mm resolution to 3.75 mm resolution isotropic (each new voxel was a composite of 30 × 30 × 30 voxels), and each voxel was assigned a unique value (Figure 1). This re-gridding factor of 30 voxels was determined empirically by performing a coarse search of down sampled voxel dimensions from 10 to 50 voxels and optimizing for least inter-subject variance. The inter-subject variance was calculated by computing the variance within each “regridded” voxel across subjects, deriving the median across voxels (as different regridding factors resulted in different number of voxels) and then calculating the mean across metrics. The labels on these re-gridded masks were defined such that each down-sampled voxel had the same unique value (Figure 2A). AFNI’s *3dROIstats* was then used to generate voxel-wise averages of the diffusion metrics and the cell counts by applying these generated masks to the both the diffusion parametric maps as well as the cell count atlas.

Correlation matrices between diffusion metrics and cell counts for each mouse were then generated by determining the Pearson correlation for each pair. The concatenated correlation was determined by calculating the Fisher Z score of all subjects’ Pearson R values and performing a one-sample *t*-test for each diffusion metric/cell count pair. To account for multiple comparisons, we applied the Holm-Sidak correction to all *p*-values, and only corrected *p*-values < 0.05 were considered significant. All statistics and modeling were performed using Python and GraphPad Prism.

### Extra Trees Prediction Pipeline

We developed a prediction pipeline to individually estimate various cell densities (Dependent variables [6]: all cells, all neurons, all glia, oligodendrocytes, astrocytes, microglia) from our diffusion metrics alone (independent variables [5]: AD, ADC, FA, RD NDI, ODI). The data from the six mice were divided into training and testing data using a sixfold Leave One Out cross-validation approach, which selected 5 mice for training and 1 mouse for testing and this was repeated 6 times for 6 non-overlap validation data sets. The model performance metrics were determined by averaging the predicted variables over the 6 trials.

These diffusion metrics are highly correlated to each other (Pines et al., 2020; Johnson et al., 2021). Since high collinearity between the independent parameters is undesirable for most prediction algorithms, the input data was first recreated by compressing the diffusion metrics into a reduced dimensional space using a Keras autoencoder (Keras, 2021; *The Python Deep Learning API*, n.d.) with the Adam optimization algorithm, optimized for mean squared error.

We then built our extra trees regression model. Model parameters were determined for each region using a grid search with a fivefold cross validation. The parameters were optimized for Pearson R rather than the slope of the fit as we were aiming for stronger relative predictability over absolute predictive power. Random decision trees were then trained on bootstrapped subsamples of the dataset over 1,000 iterations. To verify that nothing about the subsampling was driving any of the observed effects, we performed 1,000 random samplings of 70% of our data, and the resulting slopes were entirely consistent with our regression-based confidence intervals. To generate the probability distribution of our performance metrics (Pearson correlation and *p*-value), the training and testing data were randomly subsampled at 80% over 1,000 iterations for each trial prior to fitting the model (Figure 1).

## Results

### Whole Brain Diffusion Metrics Have Limited Relationships With Cell Densities

Our first question was whether the diffusion metrics and various cell counts in the whole brain had significant observable relationships. We found that the oligodendrocyte counts were negatively correlated with the AD, ADC, and RD and positively correlated with the FA and NDI. The FA was also positively correlated with total glia and astrocyte count, and the RD was negatively correlated with counts of all cells except microglia (Figure 2B).

### Whole Brain Predictor Models Fail for All Cell Types Except Oligodendrocytes

**FIGURE 3:**
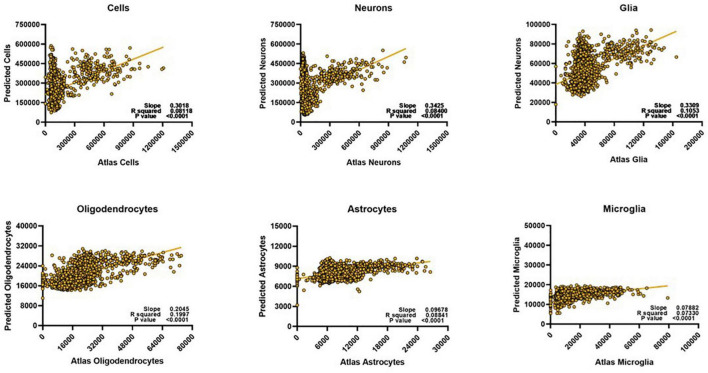
Despite optimization, whole brain voxel-wise relationships cannot be exploited to develop meaningful prediction models for most cell types. We could only successfully estimate oligodendrocyte counts for the whole brain. Predicted scale = 2× atlas scale.

We then asked whether these diffusion metric/cell count relationships were consistent enough to generate a successful prediction model for the different cell types. Since we were aiming for strong *relative* predictability, we determined model fit performance by linearly plotting the atlas counts against the predicted counts for each cell type. The stronger the Pearson correlation, the better the model was considered to be. We found that the model performed well when predicting oligodendrocyte counts (Figure 3), but that its performance on other types suffered. For cells, neurons, and glia, despite showing reliable correlation coefficients, it was evident that the model from whole-brain data could only perform well on a small subset of the voxels. When atlas counts were low, the model predicted values across a very wide range of values, providing a poor fit. For astrocytes and microglia, the fit was more consistent across the range of values (the points lie generally along the line), but the amount of variance captured is far less than for oligodendrocytes. Together, these results suggest that while our algorithm is capable of learning a set of rules for a subset of the data, a single model might not have the capacity to learn the disparate relationships between cell counts and diffusion metrics that this diversity entails. Given the diverse cellular morphologies across the brain, here is not a simple, single, relationship to map diffusion measures to cell type density that holds across the whole brain.

### Diffusion Metrics Have Unique Relationships With Different Cell Counts in a Region-Specific Fashion

**FIGURE 4:**
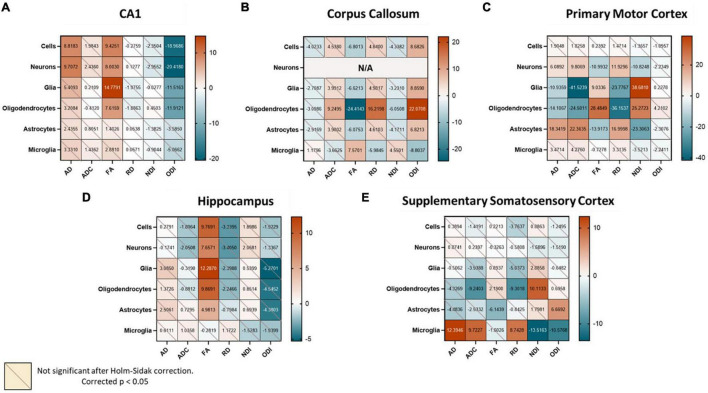
When examining different regions, we find unique region-specific relationships between diffusion metrics and cell types. Values in the correlation matrix represent the *t*-value from a one-sample *t*-test of the Z-score of the Pearson correlation coefficient of each subject’s pair. “Cells” represent counts of all cell types studied, and “Glia” is the sum of oligodendrocyte, astrocyte and microglia counts. Subplots represent correlation matrices of individual regions: **(A)** CA1, **(B)** Corpus Callosum, **(C)** Primary Motor Cortex, **(D)** Hippocampus, **(E)** Supplementary Somatosensory Cortex.

Although the complexity of the relationships between DWI and cell types may preclude a single model from predicting cell type density across the whole brain, it is still quite possible that this approach can be effective when the scope is limited to smaller regions. To further examine whether the varied cytoarchitecture of the brain influenced the nature of diffusion-metric cell count relationships, we reevaluated the correlation matrices for three representative regions of different tissue compositions: primary motor cortex (cortical gray matter), CA1 of the hippocampus (subcortical gray matter), and the corpus callosum (white matter). Indeed, each of these regions had unique relationships between cell counts and the diffusion metrics (Figures 4A–C), perhaps explaining why a common model would not be successful at predicting cell counts in these separate regions. We also observed that these relationships were distinct even to the level of subregions (Figure 4D): the overall hippocampal correlation matrix was slightly different compared to that of just the CA1 with fewer statistically significant relationships, suggesting that the subfields had enough variance in their cellular morphologies and structure to warrant separate models. Moreover, even within a given “tissue composition,” the correlation matrices did not remain consistent (Figure 4E): the primary motor area had very different significant relationships compared to the supplementary somatosensory area, despite them both being cortical regions. In the corpus callosum, correlations between the diffusion metrics and neuronal counts were not computed given the spurious number of neurons across voxels.

### Localized, Regional Models Show DWI Can Predict Cell Type Density: CA1 as a Test Case

We then asked if these stronger, region-specific relationships could be exploited to build unique models that could predict cell counts from the diffusion metrics alone. The results from the whole brain and broad regional assessments warranted the construction of a separate predictor model for every “region.” We chose the CA1 of the hippocampus as the test case to demonstrate this, given its appropriate level of cytoarchitectural complexity and size.

**FIGURE 5:**
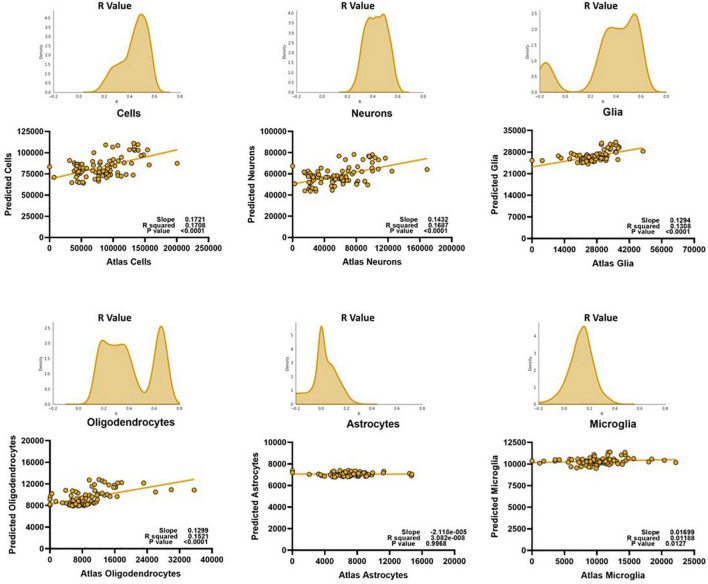
The region-specific relationships can be exploited to create models that can successfully predict certain major cell types, but not glial subtypes like astrocytes and microglia. To prevent bias, the *y*-values on the linear regressions are from an average of the predicted values of each mouse for a given voxel, on a random trial. The histograms represent the distributions of the Pearson *R* value of the model when testing and training 1,000 samplings of 80% of the data, cross-validated on all mice. Predicted scale = 2× atlas scale.

We recreated our extra trees regressor using just the CA1 data, optimized for the Pearson correlation between the atlas and predicted counts. We find that constructing our model this way results in successful prediction of cells, neurons, glia, and oligodendrocytes, but not that of astrocytes or microglia (Figure 5).

## Discussion

Here, we laid out a framework for using diffusion metrics to predict neurobiological properties of the brain, specifically cell counts. We first asked whether there were any clear voxel-wise relationships between the diffusion metrics and these various cell counts when looking at the brain as a single entity. We found that the most significant relationships were between the diffusion metrics and glial cell counts, especially those of oligodendrocytes. We also found that the tensor metrics FA and RD were highly correlated with many cell counts. However, except for oligodendrocytes, the diffusion metrics were not differentially correlated with counts of any of the cell types. In turn, our extra trees regression algorithm was only successfully able to predict counts of the oligodendrocytes from the diffusion metrics, but not of any of the other cell types. When looking at the distribution of voxel-wise counts in the major cell categories i.e., overall cell, neuron, and glia count, we found that part of the failure of the model could be caused by the cell counts themselves falling into discrete groups. We hypothesized that this was because the brain is cytoarchitecturally very complex, and it is unlikely that diffusion metrics in different parts of the brain were capturing identical microstructural properties. To further test this hypothesis, we re-evaluated the voxel-wise relationships between diffusion metrics and cell counts in separate regions representing different tissue compositions: Primary Motor Cortex (cortical gray matter), Field CA1 (subcortical gray matter), and the Corpus Callosum (white matter). We discovered that the voxel-wise diffusion metrics in these sample regions had unique relationships with their cell densities, suggesting that these metrics were indeed capturing different properties in different regions.

We next asked if modeling these regions individually would benefit our algorithm’s predictive capacity, using the CA1 of the hippocampus as a test case. Interestingly, we found that region-specific models could successfully predict all cell types studied, except for microglia and astrocytes. This is perhaps because astrocytes and microglia are slightly closer in size and shape to each other as compared to the other cells studied and our diffusion measures do not have the resolution to tell these cells apart. Moreover, these are some of the most dynamic cell types of the brain, and it has been shown that state-based morphologies could significantly influence the diffusion signal (Garcia-Hernandez et al., 2020). More studies examining the differences in the diffusion signal following acute inflammation or increase in reactivity in these cell types could refine the predictive power of our model.

There are several important caveats and limitations to the current work. First, it is important to note that the cell counts reported in this study are derived from an atlas. Though these counts are robust and reliable estimates, using atlas counts overlooks potential individual differences of the mice studied. While we do not expect differences in counts so large that they could be detected by diffusion measures, it is possible that a small part of the error from our prediction model might be arising from not being able to measure the exact cell counts of these individual mice. In addition, it is also possible that our atlas counts are more reliable for some cell types or some cell types in some regions. For example, total neuron count is unlikely to significantly vary in an adult mouse but counts of glia may well change (Pakkenberg and Gundersen, 1997; Merrill et al., 2000; Burke and Barnes, 2006). Thus, our atlas estimates may be more accurate for some cell types than for others.

Second, one could argue that the success of our model lies solely in the computational power of the extra trees prediction algorithm we utilize, or that our model might be behaving as a mere look-up table and not relying on distinct relationships between the diffusion metrics and the cell counts. However, if such were the case, a single model would have been able to predict whole brain cell counts and, as we show in Figure 3, this is not true. Moreover, the failure of the model to fit certain glial subtypes, while disappointing, demonstrates that our model is truly attempting to learn relationships between the diffusion metrics and the cell counts, and is not just fitting noise. Moreover, only the cell types that are strongly correlated with the diffusion metrics (Figures 2, 4) are successfully estimated by our model, further demonstrating that it is relying on real associations between the metrics and the counts.

It should also be noted that the model works on the same cell types when training on one hemisphere and testing on the other hemisphere of individual mice (*R* > 0.4, *p* < 0.05). Moreover, the successful results were consistent across all mice, and were consistent even in random samplings of the training and testing data, demonstrating that the prediction was not just dependent on specific mice or sets of voxels. To further verify, we also confirmed that a model trained in a region and tested in a different region was not successful, and performed at chance (*p* > 0.2), further demonstrating our model was not capable of fitting any sporadic pattern and that there were discernible region-specific relationships between the cell counts and diffusion metrics.

It should also be noted that this model was optimized for *relative accuracy*, and it grossly underestimates all cell counts, by about 50% (Figure 5). We chose to optimize for the Pearson correlation between the atlas and predicted counts over the slope of this fit as the atlas cell counts may not perfectly reflect the individual cells counts of the mice we scanned but may still follow certain spatial trends. Moreover, the clinical power of this model lies in its ability to discern between different pathological states, which are more often defined by higher or lower counts from healthy states rather than absolute thresholds.

Importantly, our pipeline only uses diffusion metrics as derived from tensor analysis and the NODDI model. We picked these methods because the tensor is still one the simplest and most popular models used for diffusion analysis; and the NODDI model leverages more complex multi-shell sequences and has previously been demonstrated to detect microstructural variance that complements the tensor metrics (Radhakrishnan et al., 2022). There are a plethora of other analysis techniques and models made possible by diffusion imaging that might be equally, if not better, suited to study gray matter cytoarchitecture non-invasively. Moreover, new models are constantly being developed, with their own advantages and failures. Determining which models generate metrics that would be most sensitive to a specific neurobiological property of interest remains a major challenge in this field. Future studies investigating how diffusion metrics derived from other analysis techniques like diffusion kurtosis imaging (Steven et al., 2014), multiple Q-shell imaging (Descoteaux et al., 2011), AxCaliber (Assaf et al., 2008), multi-tissue constrained spherical deconvolution (Nath et al., 2020), SANDI (Palombo et al., 2020) etc., perform in predicting microstructural properties will be extremely valuable.

These limitations aside, while these results demonstrate the promise of diffusion imaging in predicting explicit neurobiological properties, they also illustrate the major pitfalls of correlation studies like these. There is great interest in using non-invasive biomarkers like those found in diffusion imaging for measuring and monitoring underlying neurobiological factors. As a result, more and more studies are trying to develop such links. For the field to progress and for us to have reliable biomarkers, we will need to address a number of key challenges:

(1) Models must test a wide range of cortical regions to demonstrate regional generalizability
(2) Models must be tested not only in typical, healthy animals, but in a range of domains that present clear changes to neurobiological properties
(3) Models must adequately handle the intercorrelations between measures

### Models Must Test a Wide Range of Cortical Regions to Demonstrate Regional Generalizability

The lack of generalizability of these diffusion metrics even across brain regions of a single healthy mouse forces us to rethink how these studies are conducted. Understandably, much of the previous work focused on an individual region or on a small set of regions. This approach, while extremely valuable when trying to understand neural correlates of diffusion metrics in specific regions or contexts, cannot be generalized across the whole brain. Our results demonstrate that relationships observed in one region might not remain true in other regions and may even be completely reversed: like how we show that FA is strongly positively correlated with total glial counts in the CA1, but the same pair shows a negative relationship in the corpus callosum.

The region-specific success of our model is not particularly surprising: adjacent voxels are more likely to have similar associations between diffusion metrics and cell counts. Future correlation studies would massively benefit from region-specific models. However, we have yet to establish what properties exactly constitute a “region.” We found that simply splitting the brain into gray/white or cortical/subcortical regions is not enough to build a successful model, as our model is incapable of generalizing sufficiently. We theorize that these regions must be large enough to possess adequate variance across all metrics (we did not find significant relationships between diffusion metrics and cell counts within individual layers of the primary motor cortex), but conservative enough such that a model is not expected to learn disparate patterns of relationships (aggregating across hippocampal subfields resulted in an unreliable correlation matrix, Figure 4). Future studies that define the exact structural properties that should define the boundaries of a “region” warranting a discrete model could further help tie these two different modalities together. A major limitation of diffusion metrics is this lack of specificity and work correlating the spatial pattern of different diffusion metrics with their neurobiology will be extremely useful.

Moreover, individual cell types in different regions may have distinct morphologies that need to be accounted for to develop precise models. For example, neurons in different regions can be very structurally diverse, that could influence the diffusion signal in different ways (Palombo et al., 2016). The hippocampus, for example, has a particularly unique arrangement of neurons, which could alter diffusion metrics differently compared to even adjacent gray matter regions. Even within the hippocampus, the pyramidal cells within the CA1 and the granule cells in the dentate gyrus, while both technically neurons, could be differentially contributing to the diffusion metrics, which could explain the unreliable cell count/diffusion metric associations in the hippocampus as a whole. Likewise, cortical distributions of neurons are structurally different from those found in deeper regions like the thalamus. Other cell types, like astrocytes, come in distinct classes in different tissue types that could contribute to the diffusion signal in unique ways. While outside the scope of this paper, these morphological differences may be drastic enough to warrant splitting into different output parameters- e.g., pyramidal neurons vs. motor neurons or fibrous vs. protoplasmic astrocytes, that might improve its performance and specificity. Before this is feasible, however, more studies exploring how quantifiable variations in these different subtypes influence diffusion microstructural metrics are necessary.

Beyond just differences in cellular morphologies, local changes in brain water content caused by changes in blood flow in certain regions, spatial relationships between cells or even acute injury could significantly influence the diffusion signal (Keep et al., 2012). These associations would be harder to detect through an atlas and would benefit from more direct comparisons between specific histological properties and diffusion metrics within the same subjects.

### Models Must Be Tested Not Only in Typical, Healthy Animals, but in a Range of Domains That Present Clear Changes to Neurobiological Properties

The non-specificity of these diffusion metrics is further illustrated in our inability to anticipate what cellular changes in these diffusion metrics would translate to. Seemingly similar changes in diffusion metrics may have different anatomical implications across pathologies, age groups, and like mentioned above-even across brain regions. We found more supporting evidence for this issue of domain-specificity in a pilot study (unpublished) where we compared the diffusion signals from wild type mice (B6CBAF1/J) and mice with a CSF1R enhancer region deleted (*fmr-*intronic regulatory element or FIRE). The deletion of this region results in these mice having no microglia at all (Rojo et al., 2019). We hypothesized that this radical difference from the wild type mice would be likely reflected in their diffusion metrics as well. Surprisingly, we found that these mice had almost identical diffusion profiles throughout the brain compared to the wild type mice. Histology on these brains revealed that the absence of microglia might be compensated for by another unknown cell type, perhaps similar in morphology, and that our methods are currently incapable of telling the difference between these cell types.

This is also evident in the performance of our model: despite optimization, the slopes of the lines between atlas cell counts and predicted cell counts never get close to 1, and the intercepts never get close to 0. Our model still incorrectly predicts a significant number of cells in voxels where there are none, perhaps because the diffusion metrics cannot completely separate the contribution of different cell types to the signal. Despite the absolute prediction of cell counts suffering, however, our model still proves to be valuable as it can be represented by a linear regression and performs well when relative predictability is the determining factor of success.

### Models Must Adequately Handle the Intercorrelation Between Measures

Of note, most previous studies linking diffusion metrics with microstructural properties did so by identifying correlations separately with each diffusion metric (Table 1). Detecting these simple correlations has been extremely valuable in deciphering changes in these diffusion metrics, and their neurobiological implications. However, different patterns of cytoarchitecture could result in the exact same value for certain diffusion metrics but might not result in the exact same value for *all* diffusion metrics. Moreover, many of these diffusion metrics are highly correlated with each other, making the individual relationships challenging to interpret. As done in this paper, treating these varied diffusion metrics as a unique “signature” for each voxel may generate more specific models that can predict microarchitecture more successfully.

**FIGURE 6:**
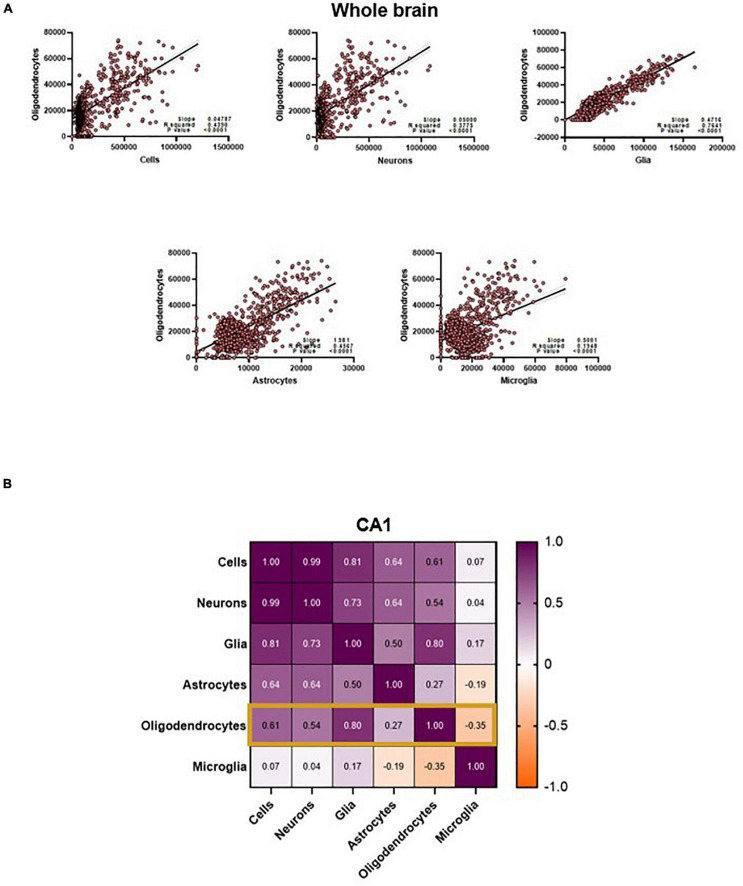
**(A)** Oligodendrocyte counts are highly correlated to other cell counts in the whole brain. **(B)** CA1 oligodendrocyte counts are only strongly correlated to counts of Cells, Neurons and total glia. Values in the matrix represent Pearson R coefficients. “Cells” represent counts of all cell types studied, and “Glia” is the sum of oligodendrocyte, astrocyte and microglia counts.

These intercorrelations exist between the neurobiological measures as well. Throughout the results in this paper, the relationship between oligodendrocytes and the diffusion metrics is curious: these cell types are the only ones that can be predicted with the whole brain data. The results in the other cells suggest that the model is not capable of generalizing patterns within the entire brain, given its cytoarchitectural complexity, but why is this not the case with the oligodendrocytes? Even if the relationships between oligodendrocytes and diffusion metrics was consistent across the whole brain, why is the cluster-like prediction structure of the other cells (Figure 3) not influencing oligodendrocyte prediction? One hypothesis is that this pertains to the relationships between the cell counts themselves. Not only are the cell types *highly correlated with each other* in the whole brain, the relationship between oligodendrocytes and neurons (and even total cells) forms a similar cluster-like pattern (Figure 6A).

Further examination shows that this interconnectedness is only true for certain cell types when examining individual regions like the CA1. One central observation is that the cell counts that are most correlated with oligodendrocyte counts in the CA1 are also the cell counts that the model predicts best (Figures 5, 6B). This might suggest that the model is primarily relying on changes in diffusion driven by oligodendrocytes to not only predict their counts, but also to predict the other cell types (given the correlation, the count of oligodendrocytes is a reasonable proxy for the count of neurons, glia, and astrocytes). While a compelling and simple hypothesis, there are aspects of our results that run against this hypothesis. Notably, our model performs best in the total cells and neuron counts instead of the oligodendrocytes. If oligodendrocytes’ diffusion properties served as a proxy for neurons, this would not be the case. One could also speculate that the mechanistic association between oligodendrocytes and myelin content could be contributing to this predictive power, and this relationship might be dominating over the contributions of smaller cells like microglia. However, this phenomenon also does not extend to other regions- even white matter regions like the corpus callosum, where the prediction of our model does not correlate with the relationship between the cell type of interest and oligodendrocytes. Moreover, it is highly unlikely that the diffusion signal is completely driven by oligodendrocyte counts. Nevertheless, these interrelationships confound our understanding of the specific neural and microstructural bases of these diffusion metrics. Central to resolving this would be future imaging studies, causally manipulating counts of these cells and breaking patterns between these cell counts. More thorough diffusion acquisition schemes, like comprehensive sampling of the diffusion space or increased b-value distribution, may also help disentangle some of these relationships.

Nevertheless, these results demonstrate that diffusion metrics in gray matter are selectively sensitive to different cell counts. Collectively, we have laid the foundation for a pipeline that could non-invasively detect cell counts in a region-specific manner. This paper further establishes that diffusion metrics can be used to examine gray matter cytoarchitecture. However, these results also raise questions on the specificity of these measures, and the extent to which these observations can be generalized. Better tools for validation, and a deliberate effort to generate large-scale publicly available datasets with multi-shelled data could not only help answer some of these questions, but also convince clinicians about the validity of this technique and be integrated in hospitals for diagnostic and other clinical applications.

## Data Availability Statement

The raw data supporting the conclusions of this article will be made available by the authors, without undue reservation.

## Ethics Statement

The animal study was reviewed and approved by University of California, Irvine, IACUC.

## Author Contributions

HR and CS: study conception, design, analysis, and interpretation of results. SS, MB-J, and AO: data collection. HR, AO, and CS: draft manuscript preparation. All authors reviewed the results and approved the final version of the manuscript.

## Conflict of Interest

The authors declare that the research was conducted in the absence of any commercial or financial relationships that could be construed as a potential conflict of interest.

## Publisher’s Note

All claims expressed in this article are solely those of the authors and do not necessarily represent those of their affiliated organizations, or those of the publisher, the editors and the reviewers. Any product that may be evaluated in this article, or claim that may be made by its manufacturer, is not guaranteed or endorsed by the publisher.

## Footnotes

1
www.mrtrix.org
